# Fulminant Food Aspiration-Induced Pneumonia Leading to Tension Pyopneumothorax after Proctectomy: An Autopsy Case

**DOI:** 10.70352/scrj.cr.25-0529

**Published:** 2026-01-29

**Authors:** Ryuta Nakao, Mizuki Honda, Nao Mitsugi, Hiroaki Nagata, Yoshinori Harada

**Affiliations:** 1 Department of Pathology and Cell Regulation, Graduate School of Medical Science, Kyoto Prefectural University of Medicine, Kyoto, Kyoto, Japan; 2 Department of Surgical Pathology, Graduate School of Medical Science, Kyoto Prefectural University of Medicine, Kyoto, Kyoto, Japan; 3 Department of Surgery, Kyoto Kizugawa Hospital, Joyo, Kyoto, Japan

**Keywords:** food aspiration, aspiration pneumonia, multiple lung abscesses, postoperative complication, rectal cancer, tension pyopneumothorax

## Abstract

**INTRODUCTION:**

Postoperative aspiration pneumonia is an uncommon but severe pulmonary complication, particularly in older adults with cognitive impairment. We report an autopsy case of fulminant aspiration pneumonia, caused by food aspiration following colorectal cancer surgery, that progressed to tension pyopneumothorax.

**CASE PRESENTATION:**

A male aged ≥75 years with dementia underwent laparoscopic high anterior resection for rectal cancer and resumed oral intake after passing a water-swallowing test. Shortly thereafter, he developed rapidly progressive pneumonia, hypoxemia, and septic shock, ultimately progressing to bilateral tension pneumothorax and death. Autopsy revealed multiple pulmonary abscesses, extensive lobular pneumonia, and subpleural fistulae in all lobes of both lungs. Grocott staining identified vegetable matter consistent with aspirated food, and colonies of *Actinomyces* species were also present, confirming aspiration pneumonia complicated by lung abscesses. Multiple pleural surface perforations from these abscesses likely caused substantial air leakage into the pleural space, culminating in uncontrolled tension pyopneumothorax. No histological signs of chronic aspiration were found.

**CONCLUSIONS:**

In older adults with dementia, massive food aspiration can lead to fatal pneumonia with progression to pyopneumothorax, even in the absence of prior aspiration history. Vigilance is essential when resuming oral intake in such patients after surgery.

## Abbreviations

ERAS
: enhanced recovery after surgery
EVG
: elastica van Gieson
H&E
: hematoxylin and eosin

## INTRODUCTION

Aspiration pneumonia is a serious postoperative complication with a mortality rate of 30%–40%.^1,2)^ With the increasing number of older adults undergoing surgery, the prevention and management of aspiration pneumonia have become essential responsibilities for surgeons. We report an autopsy case of postoperative aspiration pneumonia that progressed to tension pyopneumothorax after colorectal cancer surgery.

## CASE PRESENTATION

### Clinical summary

**Fig. 1:**
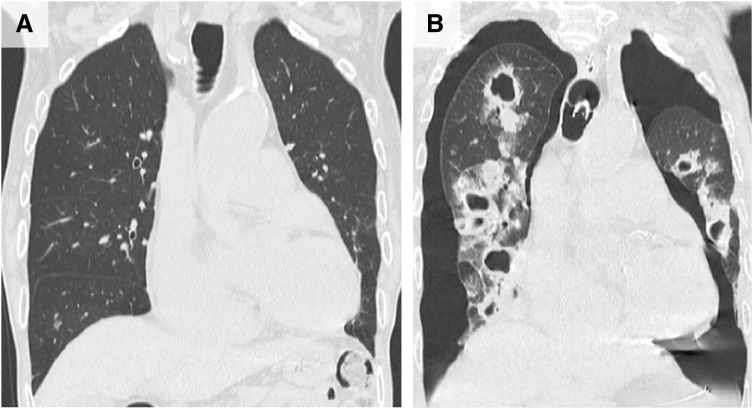
Coronal reconstructed chest CT images with lung window settings. (**A**) Preoperative CT showed no significant abnormalities in the lungs. (**B**) On POD 10, bilateral pneumothorax and multiple cavitary lesions were observed in both lung fields.

A Japanese male aged 77 years was incidentally found to have rectal wall thickening on CT performed during evaluation of a thoracic aortic aneurysm. Further investigations, including lower gastrointestinal endoscopy and thoracoabdominal CT, confirmed advanced rectal cancer without distant metastasis (clinical stage IIIA). His preoperative performance status was 2 on the Eastern Cooperative Oncology Group scale. Formal pulmonary function testing could not be performed because the patient had dementia; however, chest radiography and CT showed no findings suggestive of aspiration (**Fig. 1A**). Furthermore, preoperative arterial blood gas analysis on room air revealed a PaCO_2_ of 36 mmHg and PaO_2_ of 95 mmHg, without evidence of significant respiratory impairment. The patient was underweight (height,160 cm; weight, 45 kg; body mass index, 17.6 kg/m^2^), but his modified Glasgow Prognostic Score was 0 (serum albumin, 4.1 g/dL; C-reactive protein, 0.05 mg/dL), indicating preserved nutritional status.^3)^ Preoperative swallowing screening is not routinely performed in patients undergoing colorectal cancer surgery. Because this patient had no clinical signs of dysphagia and his nutritional status was acceptable, a formal preoperative swallowing evaluation was not requested. Specialized pre- and postoperative oral care directed by dental professionals was unavailable due to a lack of dedicated oral care staff at the hospital. Although he exhibited worsening dementia at admission, he was deemed suitable for surgery and underwent laparoscopic high anterior resection. Pathology revealed adenocarcinoma (pathological stage IIIC). Postoperatively, his vital signs were stable, and perioperative management followed the ERAS pathway.^4)^ Sedation with dexmedetomidine hydrochloride was administered only during the first postoperative night (from POD 0 to 1) to control nocturnal delirium. Postoperative pain was managed with intravenous patient-controlled analgesia and nonsteroidal anti-inflammatory drugs, and his pain remained within a tolerable range. On POD 1, he passed the water-swallowing test, and oral intake of rice gruel began on POD 2. On POD 3, he developed fever (38.6°C) and hypoxemia, prompting initiation of oxygen therapy. Chest radiography suggested pneumonia, and empiric antibiotics were started for presumed aspiration pneumonia. On POD 4, respiratory status worsened, progressing to septic shock and necessitating intubation with mechanical ventilation. On POD 5, CT revealed bilateral tension pneumothorax, and chest drains were inserted bilaterally. Multidrug-resistant *Pseudomonas aeruginosa* was detected in tracheal aspirates obtained on POD 5. Preoperative CT had shown no significant abnormalities (**Fig. 1A**), whereas POD 10 imaging demonstrated bilateral pneumothorax and multiple cavitary lesions in both lung fields (**Fig. 1B**). By POD 11, *P. aeruginosa* was also cultured from pleural effusion. Despite intensive care, his respiratory status did not improve, and he died on POD 15. The case was characterized by rapidly progressive pneumonia following rectal cancer surgery. An autopsy was performed to investigate the underlying etiology.

### Pathological examination

Autopsy was performed 10 h after death. Following macroscopic examination, all major organs were fixed in 10% buffered formalin and embedded in paraffin; tissue sections were cut at 3 μm and stained with H&E. Lung sections were additionally stained with EVG and Grocott stains.

**Fig. 2:**
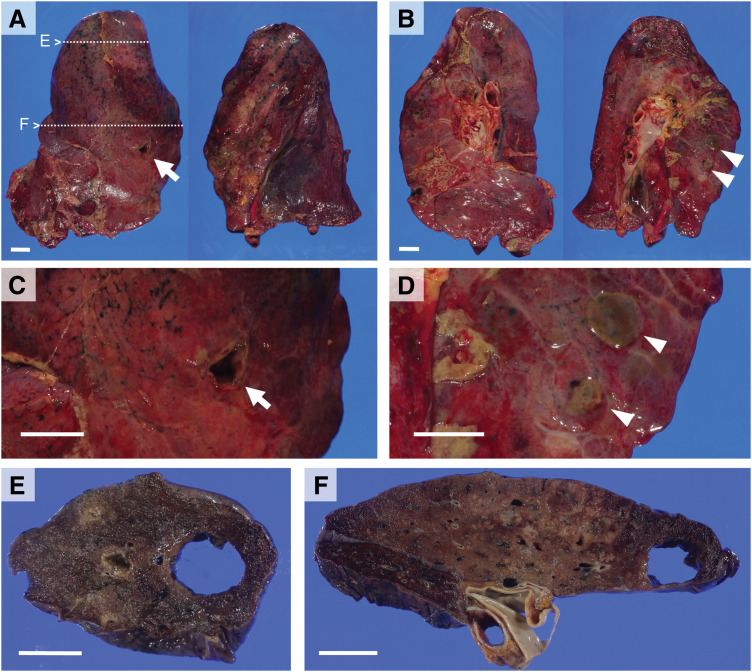
Gross findings of the lungs (right, 773 g; left, 490 g). Lateral view (**A**) and hilar view (**B**) show multiple fistulae and subpleural abscesses. A large fistula in the upper lobe of the right lung is indicated by an arrow (**A**, **C**), and subpleural abscesses in the upper lobe of the left lung are indicated by arrowheads (**B**, **D**). Cut surfaces after formalin fixation (**E**, **F**) correspond to the areas marked in the lateral view (**A**), revealing multiple pulmonary abscesses and extensive lobular pneumonia. All scale bars represent 2 cm.

Both pleural cavities contained abundant purulent effusion, and pleural surfaces were coated with extensive purulent exudate. Multiple fistulae and subpleural abscesses were present in both lungs (right, 773 g; left, 490 g) (**Fig. 2A**–**2D**). After bronchial formalin fixation, cut surfaces revealed numerous pulmonary abscesses and extensive lobular pneumonia in all lobes, predominantly in the upper lobes (**Fig. 2E** and **2F**).

**Fig. 3:**
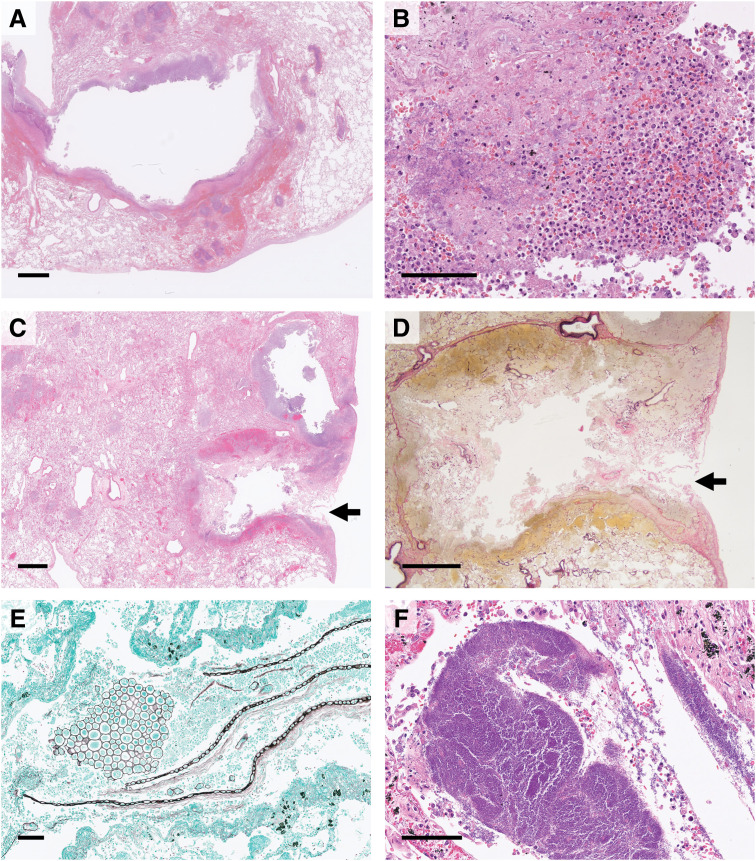
Histopathological findings of the lungs. (**A**) Low-power view of a pulmonary abscess in the upper lobe of the right lung, corresponding to **Fig. 2F**. (**B**) Abscess wall covered with purulent exudate containing neutrophils and macrophages. (**C**) Subpleural abscess ruptured through the pleura (arrow). (**D**) Higher magnification of the rupture site shown in (**C**) reveals disruption of the pleural elastic layer (arrow). (**E**) Aspirated food residues, presumably vegetable matter, were identified in all lobes with Grocott staining. (**F**) *Actinomyces* species near areas of abscess and pneumonia. Panels (**D**) and (**E**) are stained with EVG and Grocott, respectively; all other panels are stained with H&E. Scale bars represent 2 mm (**A**, **C**, **D**) and 100 μm (**B**, **E**, **F**). EVG, elastica van Gieson; H&E, hematoxylin and eosin

Histopathology showed abscess walls lined with purulent exudate containing neutrophils and macrophages (**Fig. 3A** and **3B**). Subpleural abscesses had ruptured through the pleural elastic layer, causing surface perforations measuring a few millimeters to approximately 1 cm (**Fig. 3C** and **3D**). Large quantities of aspirated material, consistent with vegetable matter, were readily identified with Grocott staining (**Fig. 3E**). Colonies of *Actinomyces* species were also observed near areas of abscess and pneumonia (**Fig. 3F**). No histological features of chronic aspiration were detected in the background lung parenchyma. Although anastomotic leakage at the rectal surgical site was present, it was considered secondary to circulatory failure from septic shock. No residual rectal cancer was identified in the examined sections. Other findings included a small, old infarct in the posterior left ventricular wall; bone marrow and other organs showed no significant abnormalities, and no underlying immunosuppressive conditions were present.

Overall, findings indicated that massive aspiration occurred soon after resumption of oral intake following rectal cancer surgery. This event led to rapidly progressive multiple lung abscesses, which caused pleural perforation, tension pyopneumothorax, and sepsis, culminating in death.

## DISCUSSION

This case represents a rare instance of fulminant aspiration pneumonia progressing to tension pyopneumothorax despite standard postoperative precautions and the absence of a history or radiological evidence of chronic aspiration.

Respiratory complications are among the most frequent postoperative events following colorectal cancer surgery.^5,6)^ Aspiration pneumonia, although relatively uncommon with an incidence of approximately 1%, is a highly severe condition with reported mortality rates of 30%–40%.^1,2,7)^ Aspiration pneumonia occurs when particulate matter, liquids, or secretions from the oral cavity, pharynx, or gastrointestinal tract enter the lungs.^8)^ In the present case, rapidly progressive bilateral pulmonary lesions developed shortly after surgery and quickly advanced to bilateral tension pneumothorax, initially raising suspicion of chemical pneumonia caused by gastric acid aspiration (Mendelson’s syndrome).^9,10)^ Mendelson’s syndrome is a sterile inflammatory reaction resulting from inhalation of acidic gastric contents, typically producing diffuse alveolar damage with hyaline membrane formation.^11)^ However, autopsy revealed lobular pneumonia and multiple lung abscesses containing food particles of presumed vegetable origin in nearly all lobes, along with *Actinomyces* colonies, confirming aspiration as the underlying cause.^12)^ The findings suggest that massive aspiration of food contaminated with *P. aeruginosa* from the oropharynx occurred upon resumption of oral intake, causing acute pneumonia and abscess formation. Multiple abscesses perforated the pleural surface, creating defects a few millimeters to approximately 1 cm in diameter. These perforations likely permitted significant air leakage into the pleural cavities, producing uncontrolled tension pyopneumothorax. This complication is extremely rare but can occur secondary to infectious pneumonia or pulmonary abscesses.^13)^ One of the unique contributions of this report is the detailed pathological clarification of tension pyopneumothorax, achieved through autopsy examination. Vegetable matter is the most frequently identified foreign material in aspiration.^14)^ Small plant fragments can be difficult to detect in inflammatory tissue using H&E staining alone; however, Grocott staining—commonly used to identify fungi—proved effective in detecting plant-derived material (**Fig. 3E**).

In this case, low body weight and dementia were clear risk factors for aspiration, yet there had been no obvious clinical signs of dysphagia before surgery, and both nutritional status and preoperative imaging findings were unremarkable. Under these circumstances, the development of severe aspiration pneumonia was difficult to predict preoperatively. Perioperative care followed the ERAS pathway, including early mobilization, early resumption of oral intake, and multimodal analgesia. Because sedation was only administered during the night of POD 0 to manage nocturnal delirium, it was considered unlikely to have had a major impact on the aspiration event that occurred on POD 2. It is possible that the progression of cognitive impairment before and after surgery was underestimated. However, because the patient actively aspirated a large volume of food, it is likely that this episode of aspiration pneumonia could only have been prevented by very close supervision and firm intervention during the first postoperative meal.

Aspiration pneumonia and lung abscesses often develop in patients with impaired consciousness, dysphagia, or immunosuppression. Although preoperative imaging revealed no signs of chronic aspiration (**Fig. 1A**), preexisting dementia and postoperative delirium represented major risk factors that should have heightened concern. The incidence of aspiration pneumonia increases with age,^7)^ and older adults with dementia are particularly susceptible to postoperative pulmonary complications after colorectal cancer surgery.^15,16)^ For patients in whom cognitive decline is anticipated, prevention of aspiration should include early postoperative reassessment of cognitive and swallowing function, combined with close supervision upon oral intake resumption. However, delaying early mobilization or postponing oral feeding conflicts with ERAS principles and may adversely affect postoperative recovery in older adults, making clinical decision-making challenging.^4)^ Furthermore, comprehensive management of nutrition, swallowing, and oral hygiene cannot be achieved by gastrointestinal surgeons alone; collaboration with co-medical professionals specializing in these areas is essential. Given the chronic shortage of healthcare workers in many developed countries, hospital- and policy-level measures may be required to ensure that high-risk older patients receive adequate multidisciplinary perioperative care.

## CONCLUSIONS

Massive aspiration shortly after resumption of oral intake following colorectal cancer surgery led to acute bilateral pneumonia and multiple lung abscesses, rapidly progressing to tension pyopneumothorax and death. Although standard precautions—such as water-swallowing testing and gradual dietary advancement—were followed, this case demonstrates that severe respiratory complications can still occur in older adults with dementia, even without prior aspiration history. Clinicians should recognize that rapidly progressive and fatal aspiration pneumonia, complicated by lung abscesses and pyopneumothorax, can occur as a rare but critical postoperative event, particularly in older adults with preexisting cognitive impairment.

## DECLARATIONS

### Availability of data and materials

Data supporting the findings of this study are available from the corresponding author upon reasonable request.
